# Persons with Diabetes' Perceptions of Family Burden and Associated Factors

**DOI:** 10.1155/2023/8015721

**Published:** 2023-01-05

**Authors:** Israel Bekele Molla, Million Abera Berhie, Kebebe Adugna Debele, Gugsa Nemera Germossa, Fikadu Balcha Hailu

**Affiliations:** Jimma University, Institute of Health, School of Nursing, Jimma, Ethiopia

## Abstract

**Background:**

Families of a person with diabetes play a vital part in diabetes management since their support helps with regimen engagement in self-management behaviors. However, focal information on the family burden of diabetes is lacking. This study is aimed at, therefore, assessing the persons with diabetes' perceptions of family burden and associated factors at a university hospital.

**Methods and Materials:**

A facility-based cross-sectional study design was conducted from July 26 to September 26, 2021on 403 persons' with diabetes attending Jimma Medical Center diabetic clinic, the study sample was selected using a simple random sampling method. The data was collected using the Zarit burden questionnaire through face-to-face interviews. Descriptive statistics (mean, standard deviation, frequency, and percentages) were ordered logistic regression, and statistical significance was declared at *P* value ≤0.05. *Results and Discussion*. About 36.8% of the patient was in mild to moderate family burden of diabetes. Farmer (AOR 5.419; CI: 1.18, 24.872), living with partners and family (AOR: 0.110, CI: 0.018, 0.659), comorbidity (AOR 5.419; CI: 1.18, 24.872), oral hypoglycemic agent (AOR: 0.380, CI: 0.191, 0.758), and being never hospitalized before because of diabetes (AOR: 0.044, CI: 0.003, 0.571) was statistically associated with a family burden.

**Conclusion:**

About one-fourth of diabetic patient-perceived mild to the moderate family burden of diabetes, persons with diabetes who work as farmers and have comorbidities have a higher opinion of family burden, whereas those who live with partners or family members, use oral hypoglycemic medications, and have never been hospitalized for diabetes have a lower view of family burden due to diabetes. The results of this study suggest that strategies for health promotion, intervention, and prevention of diabetes at the family level should consider the interaction between family member burden and the patient's sociodemographic and disease-related factors. A further large-scale study is required to validate these findings.

## 1. Introduction

Diabetes mellitus (DM) is a multifaceted disease, implying high family, social and financial cost burdens at the individual, family, community, and national levels [1–3]. Diabetes management requires lifelong complex interventions, including daily decisions concerning diet, physical activity, blood glucose monitoring, and consistent medication engagement in self-management behaviors that significantly increase the burden of diabetes on the patient, family, caregivers, health care system, and society at large [4–7]. It requires significant patient and family effort. Diabetes is thus been referred to as a “family disease” [8, 9]. Family plays a significant role in the management of diabetes [10], by providing practical support in the day-to-day management of diabetes and demanding lifelong engagement in self-management behaviors [11, 12]. The support and care given by the family for persons with diabetes at home and during visits to and admission at health facilities takes much time and creates a significant burden on the family [13, 14].

Sizeable numbers of studies show that families of PWDM are burdened physically, psychologically, emotionally, socially, and economically [15–17]. Family members are more concerned and distressed about diabetes as a severe illness than those who have diabetes themselves [5, 18]; experience different fears and worries connected with diabetes [19]; live with a constant concern for the health of the person with diabetes [20]; and experience lower positive well-being than persons with diabetes. Family members are also much concerned with the loss of family-income-associated disability, daily medications and treatment expenditures, time lost, intangible care costs, and premature deaths that create a heavy burden on the affected individuals, families, and societies [2, 21]. The psychological burden could also be higher among the partners of people with diabetes than those with diabetes themselves [22]. Interpersonal relationships, psychological functioning, and role performance of families of persons with diabetes have all been found to suffer following dealing with persons with DM. Much consumption of family income (as high as 70%) is the main economic burden reported [1–3, 6, 21, 23].

Families serve as the foundation of people's support systems in Ethiopia, with family members and extended relatives frequently depending on one another to overcome obstacles. Therefore, family members must take part in providing care and support for those who have diabetes in their family. However, there is a lack of information on diabetes family burden and how perceived family burden affects health outcomes and engagement in self-management behaviors for diabetes management. Therefore, this study is aimed at assessing the persons with diabetes' perceptions of family burden and associated factors at a university hospital.

## 2. Methods and Materials

### 2.1. Study Design and Settings

A facility-based cross-sectional study design was conducted from August to September 2021, at Jimma Medical Center (JMC). JMC is teaching and specialized hospital located in the Oromia regional State, of southwest of Ethiopia. It provides and serves approximately 15000 inpatients, 160000 outpatient attendants, and11000 emergency cases every year. The medical center has an 800-bed capacity. An estimated 640 diabetes persons visit the diabetes clinic of JMC for follow-up every month [24, 25].

### 2.2. Study Participants

From a total of 1600 adult diabetes persons registered at the diabetes clinic of JMC, a total of 403 adult persons' with diabetes 18 years or older living with family, friends, or relatives, and who have been followed up at a diabetes clinic for at least 6 months were randomly selected and included in the study. Those who have known mental health problems and are critically ill to respond were excluded from the study.

### 2.3. Data Collection

Data collection tools were prepared after reviewing relevant literature [4, 26–29]. The instrument consisted of four parts including sociodemographic characteristics, clinical factors, family-related variables, and perceived family burden. The perceived family burden questionnaire was assessed by ZARIT burden. It was validated in different parts of the world, with reported reliability and validity scores of more than 0.87 [26]. The tool was also used in African countries with a reported reliability of 0.94 in Nigeria [27] and 0.939 in Ethiopia [28]. It contains 22 items for measuring the perceived family burden of providing care and support for people with diabetes. It is assessed on a 5-point Likert scale, ranging from “0 = never” to “4 = nearly always.” Item scores are added up to give a total score ranging from 0 to 88, with higher scores indicating a greater burden [29].

#### 2.3.1. Definition of Terms and Operational Definitions

*Informal caregivers*: as individuals (e.g., spouse, parent, friend, and neighbor) who provide care to persons with diabetes, which implies the need to devote a large amount of time and effort for extended periods in tasks that are very demanding in different areas (e.g., social, emotional, and financial) [30].

*Family burden*: burden was defined as a negative impact of caring for the impaired person experienced by the caregiver on their activity (objective burden) or feeling (subjective burden) that involves emotional, physical health, social life, and financial status [31].

Perceived family burden questions are scored **0 =** Never, **1 =** Rarely, **2 =** Sometimes, **3 =** Frequently, and **4 =** Nearly Always. Scores are added up to give a total score ranging from 0 to 88. According to the original test instructions, score interpretation was as follows.

(a) 0–20: little to no burden**—**fairly low and acceptable level of burden.

(b) 21–40: mild to moderate burden**—**moderate caregiver burden sounds like a harmless, normal level of stress, but this particular stage can be the tipping point for many family caregivers.

(c) 41–60: moderate to severe burden**—**there is a growing daily responsibility, financial strain and sleep deprivation begin to add up and have more serious, longer-lasting effects on family.

(d) 61–88: severe burden**—**families are profoundly burnt out. At this point, the welfare and that of loved ones are at serious risk [29].

Data were collected through face-to-face interviews using a structured questionnaire. Family members who accompanied the persons with diabetes were asked to leave the area so that the participant can complete the study confidentially. Three senior professional nurses (one supervisor and two data collectors) were recruited to do the data collection.

To assure the quality, one day of training was given to data collectors and supervisors on the objectives of the study, data collection tools, and research ethics. In addition, supervision was conducted by supervisors and principal investigators to monitor the overall data collection process. The data collection tool was translated into local languages (Afan Oromo, and Amharic) and translated back to English to check the consistency. The questionnaire was pretested at JMC on 5% of the samples (who were later excluded from the main study), and it gives reliability (*Cronbach*′*s* Alpha = 0.989) for the ZARIT burden. The collected data were checked for completeness and consistency by the principal investigator and supervisor every day at the end of each data collection day and if necessary, corrective measures were made for the area where difficulties are identified.

### 2.4. Data Analysis

The data were entered into epi data (Manager and Entry client) 4.6 version statistical software and the generated data was exported to SPSS version 25 for analysis. In the first set of analyses, descriptive statistics were used to assess the frequency of responses for sociodemographic variables and study variables. The outcome variable was tested for normality distribution. The mean and standard deviation (SD) were reported for continuous variables. Ordered logistic regression was conducted to test the moderating effect of family burden. Statistical significance was considered at *P* value ≤0.05.

### 2.5. Ethical Considerations

An ethical clearance letter was obtained from the Institutional Review Board (IRB) of the Institute of Health of Jimma University. Then the official letter was secured from the university to the respective hospital management to gain support for the study. Before administering the questionnaires, the aims and objectives of the study were explained, and written informed consent was obtained from the study participants. The participants were also told that participation was voluntary, and confidentiality and anonymity were ensured throughout the execution of the study as participants were not required to disclose personal information on the questionnaire.

## 3. Result

### 3.1. Sociodemographic Characteristics

Out of 403 persons with diabetes sampled to be included in the study, 399 gave their responses. The results revealed that the survey included people from a variety of socioeconomic and demographic backgrounds. Participants in the study ranged in age from 18 to 85, with a mean age of 52.21 ± 15.04 years. Around two-thirds of the study participants were male, outnumbering female participants. Most of the participants in the study were married and resided in urban areas. A fifth of the participants were illiterate, and a quarter were government workers (Table 1).

### 3.2. Family-Related Characteristics

Concerning the family-related factors, the mean family monthly income was 2766.30 (SD = 2871.593), however, 26.6% (106) of the study participants reported that their monthly family income was <600 Ethiopian Birr (ETB). Regarding their living situation, 64.2% (256) of them lived with family and partners, and 62.2% (248) of them had a family history of diabetes (Table 2).

### 3.3. Diabetes Experiences

Concerning the types of diabetes, 88.5% (353) of the participants were diagnosed with T2DM (type two diabetes mellitus). The mean recent fasting blood glucose level was 162.6 mg/dL. About 45% (181) of them had diabetes for one to five years, and 62.2% (248) had additional chronic comorbidities, with hypertension being the most frequently reported one. On the other hand, 24.6% (98) reported diabetes-related complications. The average number of complications for each subject was 1.75 (SD = 0.431). Over half (53.1%) of the study participants were hospitalized because of diabetes mellitus. Regarding their mode of treatment, 42.9% (171) of them were using oral antidiabetic agents followed by 31.6% (126) on insulin (Table 3).

### 3.4. The Level of Perceived Family Burden

**Figure 1:**
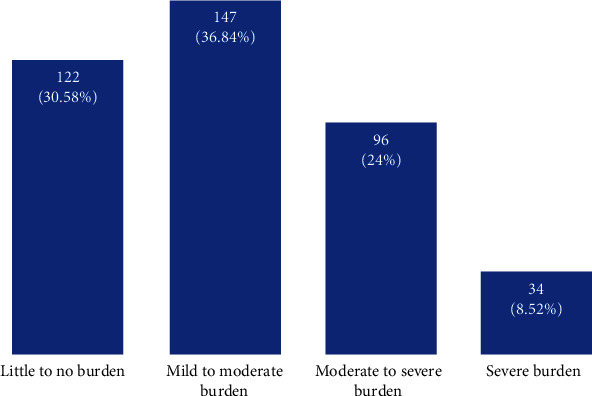
Level of persons with diabetes' perceptions of family burden and associated factors at a university hospital.

Concerning the level of family burden of care of diabetes mellitus, about 147 (36.8%) of them reported mild to moderate family burden followed by 122 (30.6%) with little to no burden, and lastly, 34 (8.5%) of them experienced severe family burden (Figure 1). The sex distribution of perceived family burden among study participants was 97 (36.6%) males were mild to moderate burden, 61 (23%) of them were moderate to severe and 30 (11.3%) were severe burden (Figure 1).

### 3.5. Sociodemographic and Disease-Related Factors with Perceived Family Burden

The odds of being in a higher level of family burden increase by a factor of 5.419 for every unit increases from independent variable unemployment as an occupation (*P* = 0.030, OR 5.419; CI: 1.18, 24.872). The odds of being in a higher level of the family burden decrease by 0.11 (*P* = 0.002, OR: 0.110, CI: 0.018, 0.659) for a unit increase in everyone living situation of living alone and by 0.185 (*P* = 0.002, OR: 0.185, CI: 0.064, 0.535) for a unit increase in everyone living situation of living alone of living with partners and family members.

The odds of having chronic diseases including hypertension, heart, and kidney diseases, as comorbidities with diabetes mellitus, the odds of being in a higher level of family burden increases by a factor of 23.18 for everyone unity increases with comorbidity of chronic diseases (*P* = 0.018, OR 5.419; CI: 1.18, 24.872).

The odds of being in the higher level of family burden decrease by 0.380 (*P* = 0.006, OR: 0.380, CI: 0.191, 0.758) for a unit increase in everyone treated with an oral hypoglycemic agent. The odds of being never hospitalized before because of diabetes-related problems indicate a decreasing probability of being in a higher level of the family burden (*P* = 0.017, OR: 0.044, CI: 0.003, 0.571) (Table 4).

## 4. Discussion

The present study revealed that 30.6%, 37.0%, 24.0%, and 8.5% of the study participants reported little, mild to moderate, moderate to severe, and severe family burden of diabetes mellitus, respectively. On the other hand, occupation, living condition, comorbidity, current treatment, and history of hospitalization were statically significant predictors of perceived family support.

In the current study, a higher proportion of study participants reported mild to moderate family burden of diabetes, whereas the study conducted in Indonesia indicated that most of the study participants reported little-no family burden and none of the family members experienced severe family burden [16], and less than study in Poland [32]. The discrepancy might be because of the cultural and social integration differences of the study population.

The odds ratio shows that the odds of being in a higher level of family burden increase by a factor of 5.419 for every unit increase in the independent variable unemployment as an occupation, which is supported by the study done in Indonesia [16]. The reason might be they are frequently engaged with their farm work and unable to control their blood glucose level, unable to maintain a healthy diet which is recommended for diabetes, and hesitant of taking their diabetic agent or insulin on time.

More than half of the study participants reported that they live with a partner and family. The odds of being in a higher level of the family burden increases by a factor of 0.185 for every unit increase in living the situation with partners and family members. This finding is in line with the findings of the study conducted in Turkey and Indonesia [16, 33]. Persons with DM stated that their families do not feel burdened when performing their care responsibilities because they consider caring for each other to be a norm as a family task.

Having diabetes mellitus with multiple comorbid conditions (hypertension, heart disease, and renal disease) raises the likelihood of having a higher level of family burden by a factor of 23. This is three times the result of the study that was conducted in China and Thailand [34, 35]. However, the result is inconsistent with the finding of the study conducted in Indonesia that found persons with diabetes mellitus had better health status when they had less of a burden on family caregivers, and vice versa with moderate correlation [16]. The difference could be explained by an increase in the complexity of care required from the patient's caregiver due to an increase in the number of DM complications, which would place a heavy burden on them.

The odds of being currently treated with an oral hypoglycemic agent indicate a decreasing probability of being in a higher level of the family burden as the values increases by a factor of 0.380. This finding is inconsistent with the finding of the study done on individuals on insulin treatment which harmed family members, causing burden, anxiety, and decreased quality of life [36–39]. The reason may be attributed to differences in the study participants, differences in the study area, and the cultural interactions of the study participants.

Our study indicated that the odds of being never hospitalized before because of diabetes-related problems indicate a decreasing probability of being in a higher level of the family burden as the value increases by a factor of 0.044. Whereas the study conducted somewhere in Nigeria indicates that the burden of diabetes correlated with the severity of illness, duration of caregiving, and coping strategies of caregivers. Family caregivers often have to contend with the emotional, social, physical, and financial strains of caregiving [40, 41].

There is a limited amount of literature regarding perceived family burden and related factors among persons with diabetes in Africa, with which to compare our study findings. This study solely depended on perceived reports from the persons with diabetes mellitus and does not distinguish between family versus friends or significant others. Because of the nature of the study design used, the cause-and-effect relationship cannot be established. This research was being studied by populations of different geographic situations, employment patterns, household structures, incomes, cultures, and ethnicity which can affect the data that researchers collect with different perspectives that each encounters every day because of the nature of the sampling method in the current study. Thus, the findings may be prone to social desirability bias. However, despite these limitations, this study was the first of its kind to be conducted on family burdens among diabetic persons in Ethiopia.

## 5. Conclusion and Recommendations

About one-fourth of the study participants reported mild to moderate family burden and only ten percent reported severe family burden. Living alone, living with partners and family, having no history of hospitalization related to diabetes, and being on oral hypoglycemic agents were significantly associated with a lower family burden of diabetes. The better the health status of people with diabetes mellitus, the lesser the burden of the family; therefore, interventions improving the health status of the patient likely will reduce the burden of the family caregiver as well is needed. The results of this study suggest that strategies for health promotion, intervention, and prevention of diabetes at the family level should consider the interaction between family member burden and the patient's sociodemographic and disease-related factors. Therefore, future researchers may conduct a longitudinal or experimental study involving both caregivers and persons to differentiate the level of burden and unmet needs of people with diabetes.

## Figures and Tables

**Table 1:** Sociodemographic characteristics of persons with diabetes' perceptions of family burden and associated factors at a university hospital (*n* = 399).

Variables	Frequency	Percent
Age		
18-29	31	7.8
30-39	64	16.0
40-49	61	15.3
50-59	72	18.0
60-69	77	19.3
70-79	84	21.1
> =80	10	2.5
Sex		
Male	265	66.4
Female	134	33.6
Marital status		
Married	319	79.9
Single	41	10.3
Divorced	19	4.8
Widowed	20	5.0
Place of residency		
Urban	249	62.4
Rural	150	37.6
Religion		
Muslim	223	55.9
Orthodox	122	30.6
Protestant	47	11.8
Catholic	7	1.8
Ethnicity		
Oromo	230	57.6
Amhara	87	21.8
Kefa	27	6.8
Gurage	7	1.8
Dawuro	29	7.3
Tigre	10	2.5
Yem	9	2.3
Occupational status		
Government employee	107	26.8
Housewife	76	19.0
Merchant	51	12.8
Farmer	90	22.6
Student	19	4.8
Daily labor	21	5.3
Pension	20	5.0
Unemployed	15	3.8
Educational status		
Illiterate	86	21.6
Read and write	24	6.0
Grade 1-4	51	12.8
Grades 5-8	61	15.3
Grade 9-12	63	15.8
College	57	14.3
University and above	57	14.3

**Table 2:** Family-related characteristics of persons with diabetes' perceptions of family burden and associated factors at a university hospital (*n* = 399).

Variables	Frequency	Percent
Level of monthly income (ETB)		
<600	106	26.6
601-1650	104	26.1
1651-3200	67	16.8
3201-5250	50	12.5
5251-7800	43	10.8
7801-10900	26	6.5
> =10901	3	0.8
Living situation		
Only with partner	64	16.0
With partner and family	256	64.2
With significant others	65	16.3
Living alone	14	3.5
Family history of diabetes		
Yes	151	37.8
No	248	62.2

**Table 3:** Diabetes clinical characteristics of persons with diabetes' perceptions of family burden and associated factors at a university hospital (*n* = 399).

Variables	Frequency	Percent
Types of DM		
Type 1	46	11.5
Type 2	353	88.5
Duration of DM		
1-5 years	181	45.4
6-10 years	146	36.6
>10 years	72	18.1
Recent fasting blood sugar level (FBS)		
<126 mg/dL	139	34.8
> =126 mg/dL	260	65.2
Presence of disability because of diabetes		
Yes	73	18.3
No	326	81.7
Presence of other chronic diseases and comorbidities		
Yes	248	62.2
No	151	37.8
Comorbidities		
Hypertension	123	30.8
Hypertension and heart disease	26	6.5
Heart disease and kidney disease	25	6.3
Heart disease	21	5.3
Kidney impairment	21	5.3
Arthritis	13	3.3
Hypertension, heart, and kidney disease	8	2.0
Osteoarthritis	4	1.0
Other	7	1.8
Mode of current treatment		
Oral diabetic agent	171	42.9
Insulin	126	31.6
Oral and insulin	102	25.6
Presence of DM complications		
Yes	98	24.6
No	301	75.4
History of hospitalization		
No hospitalization	187	46.9
Hospitalization	212	53.1

**Table 4:** Ordinal logistic regression of persons with diabetes' perceptions of family burden and associated factors at a university hospital.

Variables	B	Sig.	AOR	95% CI	
				Lower	Upper
Occupation					
Unemployed	1.690	**0.030**	5.419	1.181	24.872
Housewife	0.631	0.450	1.879	0.366	9.655
Merchant	0.850	0.255	2.339	0.541	10.112
Farmer	1.046	0.138	2.847	0.715	11.330
Student	1.610	0.065	5.003	0.903	27.708
Daily labor	1.855	0.083	6.391	0.785	52.037
Government employee	0^a^	.	1	.	.
Living situation					
Living alone	-2.211	**0.016**	0.110	0.018	0.659
Only with partner	-0.629	0.316	0.533	0.156	1.824
With partner and family	-1.685	**0.002**	0.185	0.064	0.535
With family and other partners	0^a^	.	1	.	.
Comorbidity					
Hypertension	0.740	0.480	2.095	0.269	16.309
Heart disease	0.461	0.686	1.585	0.170	14.789
Arthritis	2.132	0.075	8.428	0.805	88.276
Osteoarthritis	-1.503	0.342	0.222	0.010	4.943
Kidney impairment	0.830	0.471	2.294	0.240	21.895
Hypertension and heart disease	1.884	0.094	6.580	0.728	59.480
Heart disease and kidney disease	1.763	0.131	5.828	0.592	57.382
Hypertension, heart, and kidney disease	3.143	**0.018**	23.175	1.705	315.088
Other	0^a^	.	1	.	.
Current treatment					
Oral diabetic agent	-0.967	**0.006**	0.380	0.191	0.758
Insulin	0.539	0.211	1.715	0.737	3.988
Oral and insulin	0^a^	.	1	.	.
Hospital admission					
Never admitted	-3.113	**0.017**	0.044	0.003	0.571
One time admitted	-1.098	0.333	0.334	0.036	3.073
Two times admitted	-1.239	0.267	0.290	0.032	2.587
Three times admitted	-0.664	0.557	0.515	0.056	4.716
Four times admitted	-1.341	0.251	0.262	0.027	2.577
Five times admitted	-1.011	0.422	0.364	0.031	4.281
Six times admitted	-0.520	0.736	0.594	0.029	12.294
Seven times admitted	0.940	0.599	2.560	0.077	85.519
Eight times admitted	0^a^	.	1	.	.

^a^: constant.

## Data Availability

Data available on request.
